# Using Molecular Transmission Networks to Reveal the Epidemic of Pretreatment HIV-1 Drug Resistance in Guangxi, China

**DOI:** 10.3389/fgene.2021.688292

**Published:** 2021-09-10

**Authors:** Fei Zhang, Bingyu Liang, Xu Liang, Zhaosen Lin, Yuan Yang, Na Liang, Yao Yang, Huayue Liang, Jiaxiao Jiang, Jiegang Huang, Rongye Huang, Shanmei Zhong, Cai Qin, Junjun Jiang, Li Ye, Hao Liang

**Affiliations:** ^1^Guangxi Key Laboratory of AIDS Prevention and Treatment, School of Public Health, Guangxi Medical University, Nanning, China; ^2^Guangxi Collaborative Innovation Center for Biomedicine, Life Science Institute, Guangxi Medical University, Nanning, China; ^3^Baise Center for Disease Control and Prevention, Baise, China; ^4^Qinzhou Center for Disease Control and Prevention, Qinzhou, China

**Keywords:** antiretroviral therapy, drug resistance mutations, human immunodeficiency virus, phylogenetic analysis, pretreatment drug resistance, transmission network

## Abstract

**Introduction:**

Pretreatment drug resistance (PDR) is becoming an obstacle to the success of ART. This study investigated the prevalence of PDR and the transmission clusters (TCs) of drug resistance mutations (DRMs) in two cities where drug abuse used to be high to describe the local HIV-1 transmission dynamics.

**Methods:**

Plasma samples were obtained from 1,027 ART-naïve patients in Guangxi. Viral subtypes and DRMs were identified. Transmission network and related factors were also determined.

**Results:**

A total of 1,025 eligible sequences were obtained from Qinzhou (65.8%) and Baise (34.2%) cities. The predominant HIV-1 genotype was CRF08_BC (45.0%), followed by CRF01_AE (40.9%). The overall prevalence of PDR was 8.3%, and resistance to NNRTI was the most common. Putative links with at least one other sequence were found in 543/1,025 (53.0%) sequences, forming 111 clusters (2–143 individuals). The most prevalent shared DRMs included V106I (45.35%), V179D (15.1%), and V179E (15.1%). Clusters related to shared DRMs were more frequent and larger in CRF08_BC. The prevalence of shared DRMs increased with time, while the proportion of PDR gradually decreased. Age > 50 years was associated with clustering. Subtype CRF08_BC was more likely to have DRMs, PDR propagation, and DRM sharing.

**Conclusion:**

PDR prevalence is moderate in this region. The association between PDR and subtype CRF08_BC suggested that DRMs spreading from injection drug users (IDUs) to heterosexuals (HETs) might be the major source of PDR in this region. Our findings highlight the significance of continuous surveillance of PDR.

## Introduction

By the end of 2019, 25.4 million people living with HIV (PLWH) were accessing ART (UNAIDS, 2020). The global scale-up of ART has significantly reduced the morbidity and mortality of HIV-1. However, the resulting problem of drug resistance (DR) has also become an obstacle to eliminating the HIV/AIDS epidemic. According to the WHO (2017), transmitted drug resistance (TDR) is detected among patients without a history of antiretroviral (ARV) drug exposure, while pretreatment drug resistance (PDR) is detected among ARV drug-naïve patients initiating ART or individuals with prior ARV drug exposure initiating or re-initiating ART. In short, PDR may be transmitted at the time of infection or be acquired by virtue of prior ARV drug exposure, further leading to early virological failure (Kityo et al., 2017). Currently, PDR testing has become the standard of HIV care in many high-income countries (Hirsch et al., 2008). However, research on HIV-1 PDR transmission is rare in China, especially in southwest areas.

Genetic sequence data are increasingly being used to identify HIV-1 transmission clusters (TCs) (Wertheim et al., 2014, 2018; Chang et al., 2018), providing insights into the transmission of drug-resistant viruses (Panichsillapakit et al., 2016; Stecher et al., 2019), as molecular data allow continued surveillance of drug resistance mutations (DRMs) at baseline and interventions can be targeted at TCs with a high prevalence of DRMs. Based on this, researchers (Levintow et al., 2018) found that most TDR cases in North Carolina were identified in TCs, indicating that TDR circulates in multiple local transmission networks. Another study conducted in Croatia confirmed that transmission networks facilitated the forward transmission of drug-resistant variants (Oroz et al., 2019). In addition, German researchers clarified the same DRMs that frequently occur in genetically linked individuals, revealing the potential onward transmission of DRMs (Stecher et al., 2019). Forward transmission among ART-naïve patients was considered to be the main reason for the increasing prevalence of DR (Paraskevis et al., 2017; Bandera et al., 2019). Routine surveillance of PDR is therefore important to detect transmission networks and identify patients to enhance preventive services and promote early ART.

Currently, the prevalence of PDR is moderate in China (6.8%), while it is high in some HIV-hit regions, such as Liangshan Prefecture, Sichuan Province, China (12.2%) (Kang et al., 2020). As a high drug-use area in Southwest China, Liangshan Prefecture has recently shown a rapid increase in PDR prevalence (Liu et al., 2019), which raises an interesting issue: is high PDR associated with drug use? Studies have found that a much higher prevalence of PDR or TDR was observed among injection drug users (IDUs) compared with other populations due to many factors, including uneven access to health services, a high frequency of risk behaviors for infection and transmission (Muyldermans and Sasse, 2014), lower adherence to ART and lack of testing for baseline resistance (Liu et al., 2019).

Similar to Sichuan Province, Guangxi Province is one of the HIV-hit regions in Southwest China, where IDU was the main transmission route before 2006 (Chen et al., 2019). Previous studies among ART-naïve patients have revealed that the prevalence of TDR in Guangxi was relatively low (3.2%) during 2005–2010 (Li et al., 2014); however, the TDR prevalence in Guangxi rose to 4.6% during 2009–2013 (Zhang et al., 2015). Although TDR could provide important epidemiological information, PDR would provide more comprehensive information and would be especially useful for clinical treatment. At present, the epidemiology of PDR and the role of DRMs in the PDR genetic network have not been studied in Guangxi. In addition, drug-resistant genetic network analysis helps to elucidate the transmission characteristics of PDR, such as comparing the clustering ratio of specific DRMs to explore the impact of clustering on PDR transmission (Wertheim et al., 2017b), which provides further ideas for formulating prevention and control measures.

Presently, CRF01_AE and CRF08_BC are the main HIV-1 subtypes prevalent in Guangxi (Li et al., 2017). Qinzhou city is located in the southeast of Guangxi, and the number of cumulated HIV/AIDS cases ranks the third in the whole province (Shen et al., 2015), representing the cities with high prevalence of CRF01_AE in Guangxi. While Baise city is located in the southwest of Guangxi, and the number of newly reported HIV/AIDS cases is on the rise (Su, 2018), which represents the region in Guangxi where CRF08_BC is prevalent. Here we chose these two cities as our investigation areas to better understand the HIV-1 transmission in Guangxi. One of the main purposes of this study is to clarify the PDR prevalence as well as DRMs in this region. The second purpose is to apply genetic distance (GD)-based methods to infer local HIV-1 transmission networks, to determine the DRM transmission dynamics within network, and to explore factors related to PDR and DRM transmission.

## Materials and Methods

### Study Population

From 2015 to 2019, a total of 1,027 recently diagnosed and ART-naïve PLWH were enrolled from Qinzhou and Baise cities in Guangxi, China. Written informed consent was obtained from all participants. Blood samples were obtained and then processed in laboratory. Demographic and epidemiological information including sampling city, year of enrollment, gender, ethnicity, age, education, occupation, marital status, and transmission route were collected.

### Laboratory Testing and Subtyping

CD4 + cells were counted using FACSCalibur flow cytometer and supporting kits (BD Bioscience, United States) consistently from 2015 to 2019, which has no detection limitation. HIV-1 RNA was extracted from plasma with the High Pure Viral RNA Kit (Roche, Germany). Partial pol sequences (HXB2 position: 2,264–3,323) were amplified with the Prime Script One Step RT-PCR Kit (Takara, Dalian, China) following the procedures described in a previous study (Chen R. et al., 2018). The positive amplification replicons were purified and sequenced. The chromatogram data were cleaned and assembled using Sequencher 5.4.6. The online tool Quality Control in the Los Alamos National Laboratory HIV Database^1^ was used to rule out possible cross-contamination. All the nucleotide sequences were aligned using the online tool HIV Align (see text footnote 1) and were manually edited using BioEdit 7.0. Then, the online typing tools COMET HIV-1^2^ and HIV BLAST (see text footnote 1) were used to determine HIV-1 subtype. Discordant results were confirmed by the online tool jumping profile Hidden Markov Model (jpHMM)^3^.

### Genotypic Resistance Analysis

Drug resistance mutation screening and PDR estimation were performed according to the Genotypic Resistance Interpretation via the Stanford University HIV Drug Resistance Database HIVdb program, version 8.9^4^. DRMs were classified based on their ability to confer resistance to the NRTI, NNRTI, and PI. PDR was defined in relation to one or more of the following ARV drugs: seven NRTIs [abacavir (ABC), zidovudine (AZT), emtricitabine (FTC), lamivudine (3TC), tenofovir (TDF), stavudine (D4T), and didanosine (DDI)], five NNRTIs [doravirine (DOR), efavirenz (EFV), etravirine (ETR), nevirapine (NVP), and rilpivirine (RPV)] and eight PIs [atazanavir/r (ATV/r), darunavir/r (DRV/r), lopinavir/r (LPV/r), fosamprenavir/r (FPV/r), indinavir/r (IDV/r), nelfinavir (NFV), saquinavir/r (SQV/r), and tipranavir/r (TPV/r)]. Based on a genotypic susceptibility score, the detected PDRs were classified as high-level (score ≥ 60), intermediate (score 30–59), or low-level (score 15–29).

### Genetic Network Inference

The pairwise Tamura-Nei 93 (TN93) GD was calculated for all the sequences and the three predominant subtypes (CRF08_BC, CRF01_AE, and CRF07_BC) using HYPHY 2.2.4. To obtain a high-resolution molecular network, GD threshold for all sequences and three major subtypes were optimized to identify the largest number of molecular clusters, avoid forming giant clusters, and find out more potential transmission relationships (Wertheim et al., 2017a). The optimal GD threshold was defined as the distance that identifies the maximum number of TCs. The results showed that 0.015 was the optimal GD among the subtypes, and 0.016, 0.012, and 0.014 were the optimal GDs for CRF01_AE, CRF08_BC, and CRF07_BC, respectively. The HIV-1 genetic network was visualized and analyzed using Cytoscape 3.8.0. Shared DRM was defined as the presence of any same DRM in two genetically linked individuals. A PDR-related cluster was defined as one that contains three or more identical DRMs. Large TCs were defined as clusters containing 10 or more individuals.

### Statistical Analysis

Demographic and epidemiological information were examined to identify missing data and errors. All categorical variables were summarized into quantities and proportions. Chi-square and Fisher’s exact tests were used to compare differences between groups. Factors associated with DRMs, PDR, clustering, and shared DRM were evaluated by logistic regression analyses. All the independent variables of the univariable logistic regression analysis were incorporated into the multivariable logistic regression model. The crude OR, adjusted OR, and 95% CI were calculated. And missing covariables were automatically excluded during logistic regression analyses. The *E*-value package in R software was calculated to evaluate the potential impact of unmeasured confounders. The Cochran-Armitage trend analysis was used to assess the trend of DRMs, PDR, and shared DRM in TCs. All statistical analyses were performed using IBM SPSS Statistics 26.0. *P* values were two-sided with a significance level of 0.05.

## Results

### Characteristics of the Study Population

Partial pol sequences were obtained from 1,025 participants, including 736 (71.8%) males and 289 (28.2%) females (Table 1). Among them, 216 (21.1%) were recruited in 2015–2016, 206 (20.1%) in 2017, 322 (31.4%) in 2018, and 281 (27.4%) in 2019. 56.1% of the individuals were married, and 90.4% of them had a junior high school-level education or below. A total of 66.4% of the participants were of Han ethnicity and 36.0% were over 50 years old. 77.4% of the participants were infected via heterosexuals (HETs), followed by IDUs (18.7%). 69.3% of participants were farmers. A total of 850 patients (84.0%) had CD4 + cell counts < 499 cells/μl. Of note, data on ethnicity, education, occupation, and CD4 + cell counts were missing for 4, 7, 2, and 14 patients, respectively.

**TABLE 1:** Factors associated with drug resistance mutation (DRM) and clustering among HIV-1 infected and ART-naïve individuals in Guangxi, 2015–2019.

**Characteristic**	**Total *n* (%)**	**DRM**				**Clustering**			
		***n* (%)**	***P* ^a^**	**COR (95% CI)**	**AOR (95% CI)**	***n* (%)**	***P* ^a^**	**COR (95% CI)**	**AOR (95% CI)**
All	1025 (100)	217 (21.2)				543 (53.0)			
Sampling city			0.552				0.603		
Qinzhou	674 (65.8)	139 (20.6)		1	1	361 (53.6)		1	1
Baise	351 (34.2)	78 (22.2)		1.100 (0.804-1.504)	0.768 (0.350-1.686)	182 (51.9)		0.934 (0.721-1.209)	0.885 (0.459-1.706)
Year of enrolment			0.086				0.114		
2015-2016	216 (21.1)	55 (25.5)		1	1	129 (59.7)		1	1
2017	206 (20.1)	32 (15.5)		0.538 (0.331-0.875)*	0.794 (0.455-1.385)	100 (48.5)		0.636 (0.433-0.935)*	0.486 (0.309-0.763)*
2018	322 (31.4)	67 (20.8)		0.769 (0.512-1.156)	0.993 (0.616-1.601)	165 (51.2)		0.709 (0.500-1.005)	0.657 (0.437-0.987)*
2019	281 (27.4)	63 (22.4)		0.846 (0.559-1.281)	1.074 (0.489-2.357)	149 (53.0)		0.761 (0.532-1.090)	0.925 (0.474-1.805)
Subtype			**<0.001**				**0.004**		
CRF01_AE	419 (40.9)	59 (14.1)		1	1	231 (55.1)		1	1
CRF08_BC	461 (45.0)	125 (27.1)		2.270 (1.610-3.200)*	2.349 (1.593-3.463)*	255 (55.3)		1.007 (0.772-1.314)	1.014 (0.745-1.381)
CRF07_BC	91 (8.9)	13 (14.3)		1.017 (0.532-1.945)	1.041 (0.534-2.028)	38 (41.8)		0.584 (0.369-0.923)*	0.589 (0.363-0.954)*
Others	54 (5.2)	20 (30.7)		3.589 (1.936-6.653)*	3.320 (1.733-6.360)*	19 (35.2)		0.442 (0.245-0.798)*	0.543 (0.291-1.011)
Gender			0.711				0.878		
Male	736 (71.8)	158 (21.5)		1	1	391 (53.1)		1	1
Female	289 (28.2)	59 (20.4)		0.938 (0.671-1.313)	1.235 (0.827-1.846)	152 (52.6)		0.979 (0.745-1.286)	0.868 (0.628-1.198)
Ethnic			0.068				0.233		
Han	681 (66.4)	139 (20.4)		1	1	371 (54.6)		1	1
Zhuang	326 (31.8)	78 (23.9)		1.219 (0.889-1.671)	1.496 (0.864-2.591)	160 (48.9)		0.798 (0.613-1.039)	0.676 (0.433-1.057)
Others	14 (1.4)	0 (0)		0 (0)		8 (57.1)		1.111 (0.381-3.235)	0.977 (0.307-3.110)
Age (years)			**0.004**				**<0.001**		
=30	128 (12.5)	36 (28.1)		1	1	54 (42.2)		1	1
31-40	291 (28.4)	73 (25.1)		0.856 (0.536-1.366)	0.843 (0.497-1.430)	144 (49.5)		1.342 (0.883-2.042)	1.340 (0.840-2.138)
41-50	237 (23.1)	51 (21.5)		0.701 (0.427-1.149)	0.716 (0.405-1.265)	114 (48.1)		1.270 (0.823-1.959)	1.257 (0.769-2.055)
>50	368 (36.0)	57 (15.5)		0.468 (0.290-0.755)*	0.571 (0.315-1.033)	230 (62.5)		2.284 (1.517-3.439)*	2.251 (1.366-3.710)*
Education			0.726				0.125		
Illiteracy	89 (8.7)	15 (16.9)		1	1	48 (53.9)		1	1
Primary school	486 (47.7)	104 (21.4)		1.343 (0.740-2.437)	1.140 (0.590-2.203)	271 (55.8)		1.077 (0.684-1.695)	1.179 (0.714-1.947)
Junior high school	345 (34.0)	77 (22.3)		1.417 (0.770-2.609)	1.119 (0.564-2.219)	179 (51.9)		0.921 (0.577-1.470)	1.155 (0.681-1.958)
Middle high school and above	98 (9.6)	20 (20.4)		1.265 (0.603-2.654)	1.153 (0.495-2.683)	42 (42.9)		0.641 (0.359-1.142)	0.775 (0.401-1.499)
Occupation			0.445				0.133		
Others	314 (30.7)	62 (19.7)		1	1	155 (49.4)		1	1
Farmer	709 (69.3)	155 (21.9)		1.137 (0.818-1.582)	1.154 (0.782-1.703)	386 (54.4)		1.226 (0.940-1.600)	0.918 (0.669-1.258)
Marital status			**0.003**				**0.008**		
Unmarried/cohibiting	257 (25.1)	72 (28.0)		1	1	118 (45.9)		1	1
Married	573 (56.1)	114 (19.9)		0.638 (0.454-0.897)*	0.677 (0.445-1.029)	327 (57.1)		1.566 (1.165-2.105)*	1.287 (0.898-1.843)
Divorced/widowed	192 (18.8)	30 (15.6)		0.476 (0.296-0.765)*	0.523 (0.304-0.900)*	96 (50.0)		1.178 (0.810-1.713)	0.910 (0.587-1.410)
Transmission route			0.145				0.066		
HETs	793 (77.4)	156 (19.7)		1	1	429 (54.1)		1	1
IDUs	192 (18.7)	52 (27.1)		1.517 (1.055-2.181)*	0.928 (0.557-1.548)	96 (50.0)		0.848 (0.619-1.163)	0.796 (0.514-1.232)
MSM	19 (1.9)	4 (21.1)		1.089 (0.356-3.326)	0.721 (0.204-2.543)	5 (26.3)		0.303 (0.108-0.849)*	0.738 (0.239-2.285)
Others/NA	21 (2.0)	5 (23.8)		1.276 (0.460-3.536)	0.881 (0.247-3.137)	13 (61.9)		1.379 (0.565-3.363)	1.910 (0.622-5.866)
CD4+ cell count (cells/ul)			0.731				0.705		
<200	419 (41.4)	87 (20.8)		1	1	229 (54.7)		1	1
200-499	431 (42.6)	90 (20.9)		1.007 (0.723-1.403)	0.816 (0.566-1.176)	225 (52.2)		0.906 (0.692-1.187)	0.987 (0.734-1.328)
=500	161 (16.0)	38 (23.6)		1.179 (0.764-1.819)	1.012 (0.624-1.642)	83 (51.6)		0.883 (0.613-1.271)	0.994 (0.664-1.486)

*^*a*^Chi-square and Fisher’s exact test. Statistically significant *P* values are indicated in bold. Of 1025 participants, data on ethnicity, education, occupation and CD4+ cell counts are missing for 4, 7, 2 and 14 patients, respectively. Abbreviation: DRM, drug resistance mutation; COR, crude odds ratio; AOR, adjusted odds ratio; CI, confidence interval; HETs, heterosexuals; IDUs, injection drug users; MSM, man who have sex with man; NA, not available.*

* indicates that the P value in logistic regression analysis is less than 0.05.

### HIV-1 Genotypes Distribution

In this study, CRF08_BC was the predominant HIV-1 genotype and accounted for 45.0% (461/1,025) of cases, followed by CRF01_AE (40.9%, 419/1,025) and CRF07_BC (8.9%, 91/1,025). In addition, 54 patients were infected with other HIV-1 subtypes, including subtypes B (*n* = 1), C (*n* = 9), G (*n* = 1), CRF57_BC (*n* = 2), CRF55_01B (*n* = 12), and CRF59_01B (*n* = 2) and unique recombinant forms (URFs) (*n* = 27). The sequences from Baise city were subtyped as CRF08_BC (51.0%, 179/351), CRF01_AE (33.9%, 119/351), CRF07_BC (8.5%, 30/351), and others (6.6%, 23/351), and the sequences from Qinzhou city were subtyped as CRF01_AE (44.5%, 300/674), CRF08_BC (41.8%, 282/674), CRF07_BC (9.1%, 61/674), and others (4.6%, 31/674). The different distribution of HIV-1 subtypes between Baise and Qinzhou cities was statistically significant (*P* = 0.006).

### Prevalence of Pretreatment HIV-1 DR

**FIGURE 1:**
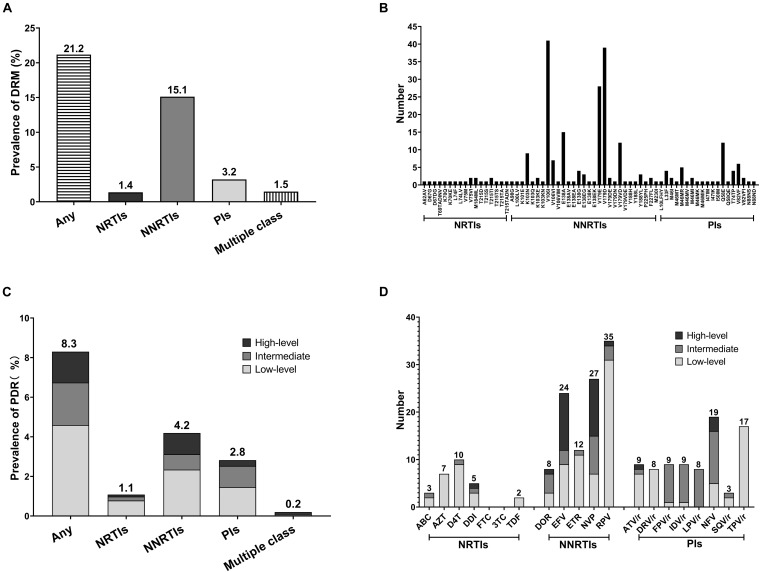
Prevalence and frequency of HIV-1 drug resistance mutation (DRM) and pretreatment drug resistance (PDR) in two severe drug abuse regions in Guangxi, 2015–2019. **(A)** HIV-1 DRM prevalence by drug class. **(B)** Frequency of NRTIs, NNRTIs, and PIs DRMs. **(C)** HIV-1 PDR prevalence by drug class and drug resistance (DR) level. **(D)** Frequency of HIV-1 PDR to 20 antiretroviral (ARV) drugs.

The prevalence of any DRMs among the 1,025 participants was 21.2% (217/1,025) (Figure 1A) and remained stable from 2015 to 2019 [2015–2016: 25.5% (55/216), 2017: 15.5% (32/206), 2018: 20.8% (67/322), 2019: 22.4% (63/281); and *P* = 0.086] (Table 1). NNRTI and NRTI resistance mutations were detected in 155/1,025 (15.1%) and 14/1,025 (1.4%) individuals, respectively (Figure 1A). Totally, 33 individuals (3.2%) had at least one DRM to PI. Furthermore, six individuals (0.6%) harbored both NNRTI and NRTI resistance mutations, nine individuals (0.9%) harbored both NNRTI and PI mutations. V106I (4.0%, 41/1,025) and V179D (3.8%, 39/1,025) were the most common DRMs, followed by V179E (2.7%, 28/1,025) (Figure 1B). All three of the most common DRMs were NNRTI-related. The frequency of DRMs was different between groups (subtype, age, and marital status) (Table 1).

The prevalence of PDR was 8.3% (85/1,025), with 4.2% (43/1,025) for NNRTI, 1.1% (11/1,025) for NRTI and 2.8% (29/1,025) for PI (Figure 1C). PDR was concentrated in NNRTI. Moreover, two individuals had dual-class resistance (1 NNRTI + NRTI and 1 NNRTI + PI). No triple-class resistance was found. PDR to single drug was highest for RPV (3.4%, 35/1,025), NVP (2.6%, 27/1,025), and EFV (2.3%, 24/1,025), while lowest for SQV/r (0.3%, 3/1,025), ABC (0.3%, 3/1,025), and TDF (0.2%, 2/1,025) (Figure 1D). High-level resistance was most common in EFV (75%, 12/16) and NVP (75%, 12/16). The resistance levels and frequencies of three different classes of ARV drugs are shown in the Figure 1D.

### Pretreatment Drug Resistance Transmission Within the Genetic Network

**FIGURE 2:**
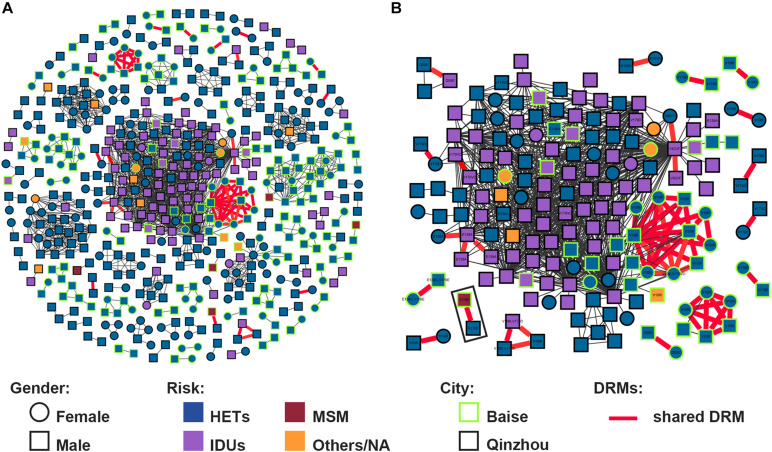
HIV-1 transmission clusters (TCs) of PDR in two severe drug abuse regions in Guangxi, 2015–2019. **(A)** All clusters are shown. **(B)** Enlargement of clustering individuals harboring shared DRM labeled with each node. Squares and circles denote male and female. Nodes color indicates the reported transmission route and the frame color indicates the sampling city. All edges represent a genetic distance (GD) of <1.5%. Edges in bold red indicate individuals who shared DRM.

**FIGURE 3:**
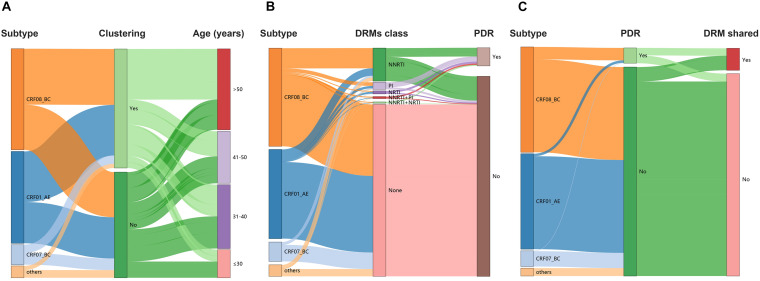
HIV-1 TCs of PDR for three major subtypes. **(A)** Genetic network for CRF01_AE and all edges represent a GD of <1.4%. **(B)** Genetic network for CRF08_BC and all edges represent a GD of <1.2%. **(C)** Genetic network for CRF07_BC and all edges represent a GD of <1.6%. Squares and circles denote male and female. Nodes color indicates the reported transmission route and the frame color indicates the sampling city. Edges in bold red indicate individuals who shared DRM. And shared DRM were labeled with each node.

**FIGURE 4:**
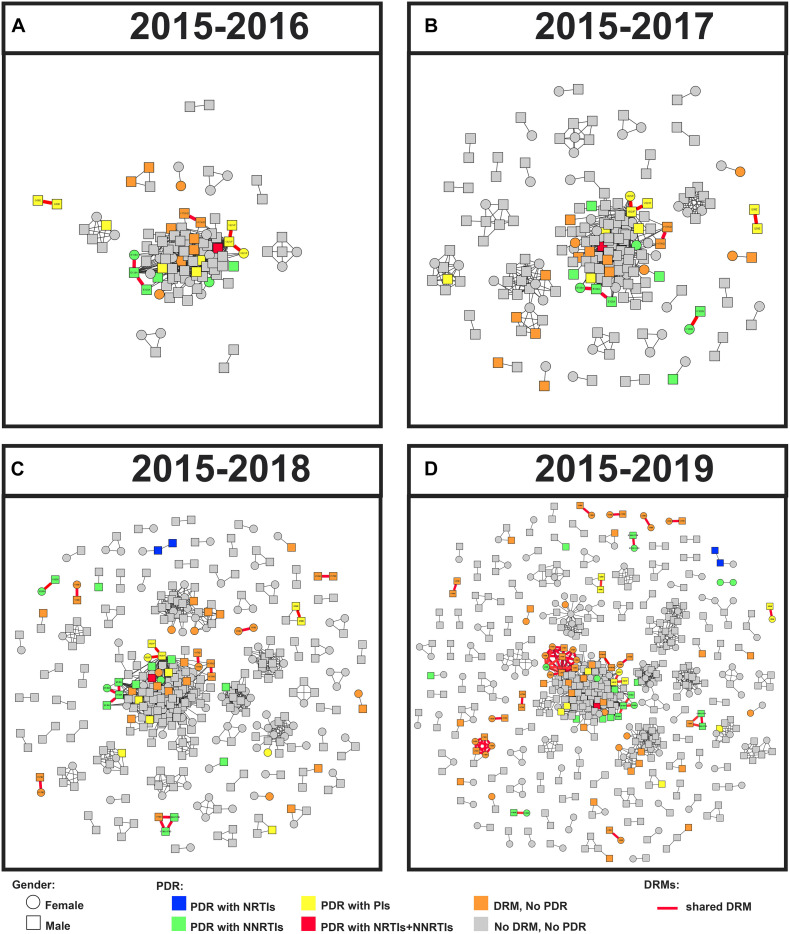
Linked DRM and PDR, and cluster growth of the HIV-1 genetic network in Guangxi, 2015–2019. Squares and circles denote male and female. Nodes were colored by the presence of HIV-1 DRM and PDR. All edges represent a GD of < 1.5%. Edges in bold red indicate individuals who shared DRM. And shared DRM were labeled with each node. Annual growth of the genetic network is shown from panels **(A–D)**.

Putative transmission links with at least one other sequence were found for 543/1,025 (53.0%) sequences, forming 111 clusters (2–143 individuals) (Figure 2A). And 7 (6.3%, 7/111) clusters were identified as large TCs. The prevalence of PDR was not different between clustering and non-clustering individuals [6.8% (37/543) vs. 10.0% (48/482); *P* = 0.068]. Clustering individuals were less likely to include participants recruited in 2017 (AOR = 0.486, 0.309–0.763) and 2018 (AOR = 0.657, 0.437–0.987) and less likely to be subtype CRF07_BC (AOR = 0.589, 0.363–0.954) (Table 1). Individuals > 50 years old were more likely to cluster (AOR = 2.251, 1.366–3.710) (Figure 3A). Of the 217 sequences harboring DRMs, 100 (46.1%) were identified as members of 36 different clusters (Figure 4D). Subtype CRF08_BC (COR = 2.270, 95% CI = 1.610–3.200; AOR = 2.349, 95% CI = 1.593–3.463) and others (COR = 3.589, 95% CI = 1.936–6.653; AOR = 3.320, 95% CI = 1.733–6.360) were more likely to have DRMs than subtype CRF01_AE (Figure 3B). The lower limit of *E*-value of age > 50 years old, CRF08_BC and DRM were 1.613 and 1.837, respectively (Supplementary Table 1). Among clustering individuals with DRMs, 53/100 (53.0%) were genetically linked partners in 15 DRM sharing clusters, suggesting DRM transmission among ART-naïve patients (Figure 2B). The most prevalent shared DRM was V106I (45.35%, 24/53), followed by V179D (15.1%, 8/53), and V179E (15.1%, 8/53). In particular, two TCs contained shared DRMs of E138G + V179E and V106I + V179D in Baise and Qinzhou cities, respectively. The largest PDR-related cluster within network contained DRM propagation of E138A, V82VF, V179D, V179VD, and V106I, while the remaining two PDR-related clusters are only involved in V106I propagation. More remarkably, a man who have sex with man (MSM) from Baise city and a male HET from Qinzhou city comprised a transmission network, both of which were subtype CRF55_01B, showing DRM sharing for V179E.

### Subtype and Dynamic Transmission Network

**FIGURE 5:**
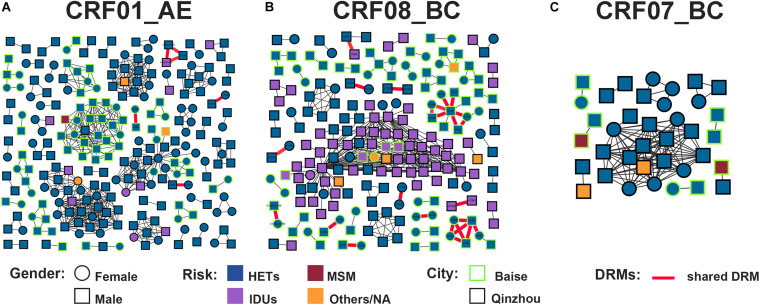
Sankey plots. **(A)** Lineage dispersal relation between subtype, clustering, and age among all individuals. **(B)** Lineage dispersal relation between subtype, DRMs class, and PDR among all individuals. **(C)** Lineage dispersal relation between subtype, PDR, and DRM shared among clustering individuals within networks. DRM, drug resistance mutation; PDR, pretreatment drug resistance; NA, not available.

Under the optimal GD threshold, transmission network analysis found 242/419 (57.8%) genetically linked individuals forming 53 clusters (2–30 individuals) for CRF01_AE (Figure 5A). DRM sharing appeared among nine individuals (two from Baise city and seven from Qinzhou city) in four different clusters. Furthermore, 201/461 (43.6%) sequences generated 49 clusters (2–73 individuals) for CRF08_BC (Figure 5B). Of those, 32 individuals (20 from Baise city and 12 from Qinzhou city) in twelve clusters developed DRM sharing. As shown, clusters related to shared DRMs were more frequent and larger in CRF08_BC than in CRF01_AE (Figures 5A,B and Table 1). Regarding CRF07_BC, putative transmission links were found for 37/91 (40.7%) sequences, forming 10 clusters (2–16 individuals) (Figure 5C). However, no observed shared DRMs were identified.

The annual growth of the transmission network is shown in Figure 4. The prevalence of DRMs among clustering and non-clustering individuals was relatively stable (Table 2). Although there was a decline in 2017, the ratio of shared DRMs generally increased (0.10, 0.04, 0.08, and 0.20 for 2015–2016, 2017, 2018, and 2019, respectively; P for trend = 0.027), indicating that 9.3, 4.0, 7.3, and 16.8% of patients shared DRMs among clustering individuals participating in 2, 3, 8, and 15 clusters in 2015–2016, 2017, 2018, and 2019, respectively. In contrast, despite the increase in 2018, the proportion of PDR generally decreased among clustering individuals (Table 2). There was no significant change in PDR among non-clustering individuals (Table 2).

**TABLE 2:** Prevalence of DRM, pretreatment drug resistance (PDR), and shared DRM among clustering and non-clustering individuals from 2015 to 2019 (%).

Variables		Total (*n* = 1,025)	2015–2016 (*n* = 216)	2017 (*n* = 206)	2018 (*n* = 322)	2019 (*n* = 281)	*P* for χ^2^	*P* for trend	OR (95% CI)
DRM	Clustering	18.4	24.0	13.0	13.3	22.8	**0.022**	0.805	0.976 (0.805–1.184)
	Non-clustering	24.3	27.6	17.9	28.7	22.0		0.835	0.979 (0.805–1.192)
	All	21.2	25.5	15.5	20.8	22.4	0.086	0.811	0.983 (0.858–1.128)
PDR	Clustering	6.8	14.0	4.0	4.8	4.7	0.068	**0.005**	0.648 (0.477–0.880)
	Non-clustering	10.0	14.9	5.7	14.0	5.3		0.142	0.812 (0.614–1.073)
	All	8.3	14.4	4.9	9.3	5.0	**<0.001**	**0.004**	0.740 (0.604–0.907)
shared DRM	Clustering	9.8	9.3	4.0	7.3	16.8	NA	**0.027**	1.355 (1.036–1.772)

*Statistically significant *P* values are indicated in bold. DRM, drug resistance mutation; PDR, pretreatment drug resistance; OR, odd ratio; CI, confidence interval; NA, not available.*

### Factors Associated With HIV-1 PDR and Shared DRM

The risk factors associated with HIV-1 PDR and shared DRM among clustering individuals are listed in Table 3. The distribution of PDR by year of patient enrollment, subtype, age, and transmission route indicate that they were significantly different. In univariate logistic regression analysis, year of enrollment, subtype, and transmission route were significantly associated with PDR. PDR among clustering individuals was less frequent in 2017 (COR = 0.257, 95% CI = 0.084–0.785), 2018 (COR = 0.314, 95% CI = 0.132–0.748), and 2019 (COR = 0.304, 95% CI = 0.123–0.753) than in 2015–2016. However, this significance was lost in multivariate logistic regression analysis. The proportion of IDUs among participants harboring PDR in TCs was slightly higher in clustering individuals (40.5 vs. 35.4%; COR = 3.426, 95% CI = 1.704–6.887), but this significance was lost after adjusting for all variables. Subtype CRF08_BC (COR = 5.000, 95% CI = 2.041–12.246; AOR = 4.083, 95% CI = 1.498–11.127) was more likely to occur PDR (Figure 3C). The lower limit of *E*-value of CRF08_BC and PDR propagation within network was 1.747 (Supplementary Table 1).

**TABLE 3:** Factors associated with PDR and shared DRM among individuals within clusters in HIV-1 genetic network.

Characteristic	PDR				Shared DRMs			
	*n* (%)	*P* ^a^	COR (95% CI)	AOR (95% CI)	*n* (%)	*P* ^a^	COR (95% CI)	AOR (95% CI)
All (*n* = 543)	37 (6.8)				53 (9.8)			
Sampling city		0.220				**0.012**		
Qinzhou (*n* = 361)	28 (7.8)		1	1	27 (7.5)		1	1
Baise (*n* = 182)	9 (4.9)		0.619 (0.286–1.340)	0.548 (0.065–4.628)	26 (14.3)		2.062 (1.165–3.650)*	0.154 (0.013–1.789)
Year of enrollment		**0.003**				**0.004**		
2015–2016 (*n* = 129)	18 (14.0)		1	1	12 (9.3)		1	1
2017 (*n* = 100)	4 (4.0)		0.257 (0.084–0.785)*	0.419 (0.119–1.484)	4 (4.0)		0.406 (0.127–1.300)	0.657 (0.177–2.440)
2018 (*n* = 165)	8 (4.8)		0.314 (0.132–0.748)*	0.508 (0.185–1.393)	12 (7.3)		0.765 (0.332–1.764)	1.245 (0.463–3.352)
2019 (*n* = 149)	7 (4.7)		0.304 (0.123–0.753)*	0.444 (0.063–3.105)	25 (16.8)		1.966 (0.944–4.092)	7.051 (0.683–72.817)
Subtype		**<0.001**				**<0.001**		
CRF01_AE (*n* = 231)	6 (2.6)		1	1	7 (3.0)		1	1
CRF08_BC (*n* = 255)	30 (11.8)		5.000 (2.041–12.246)*	4.083 (1.498–11.127)*	44 (17.3)		6.673 (2.941–15.142)*	8.641 (3.475–21.490)*
CRF07_BC (*n* = 38)	1 (2.6)		1.014 (0.119–8.661)	1.095 (0.120–10.018)	0 (0)		0 (0)	0 (0)
Others (*n* = 19)	0 (0)		0 (0)	0 (0)	2 (10.5)		3.765 (0.725–19.544)	2.807 (0.442–17.828)
Gender		0.892				**0.021**		
Male (*n* = 391)	27 (6.9)		1	1	31 (7.9)		1	1
Female (*n* = 152)	10 (6.6)		0.949 (0.448–2.012)	1.175 (0.453–3.049)	22 (14.5)		1.965 (1.098–3.517)*	2.168 (0.983–4.781)
Ethnic		0.917				**0.010**		
Han (*n* = 371)	27 (7.3)		1	1	28 (7.5)		1	1
Zhuang (*n* = 160)	10 (6.3)		0.849 (0.401–1.799)	2.292 (0.513–10.232)	25 (15.6)		2.269 (1.277–4.031)*	2.278 (0.728–7.129)
Others (*n* = 8)	0 (0)		0 (0)	0 (0)	0 (0)		0 (0)	0 (0)
Age (years)		**0.045**				0.273		
≤ 30 (*n* = 54)	2 (3.7)		1	1	5 (9.3)		1	1
31–40 (*n* = 144)	17 (11.8)		3.480 (0.776–15.602)	2.696 (0.525–13.843)	20 (13.9)		1.581 (0.562–4.446)	2.035 (0.556–7.444)
41–50 (*n* = 114)	7 (6.1)		1.701 (0.341–8.476)	1.503 (0.258–8.773)	10 (8.8)		0.942 (0.306–2.905)	1.045 (0.256–4.272)
> 50 (*n* = 230)	11 (4.8)		1.306 (0.281–6.071)	2.297 (0.370–14.272)	18 (7.8)		0.832 (0.295–2.350)	1.278 (0.307–5.321)
Education		0.177				0.572		
Illiteracy (*n* = 48)	2 (4.2)		1	1	7 (14.6)		1	1
Primary school (*n* = 271)	19 (7.0)		1.734 (0.391–7.699)	1.287 (0.230–7.197)	24 (8.9)		0.569 (0.230–1.406)	0.768 (0.228–2.587)
Junior high school (*n* = 179)	16 (8.9)		2.258 (0.501–10.179)	1.586 (0.274–9.171)	19 (10.6)		0.696 (0.274–1.766)	0.950 (0.272–3.323)
Middle high school and above (*n* = 42)	0 (0)		0 (0)	0 (0)	3 (7.1)		0.451 (0.109–1.867)	0.987 (0.174–5.598)
Occupation		0.175				**0.048**		
Others (*n* = 155)	30 (7.8)		1	1	44 (11.4)		1	1
Farmer (*n* = 386)	7 (4.5)		1.782 (0.766–4.147)	1.460 (0.542–3.934)	9 (5.8)		2.087 (0.993–4.386)	2.076 (0.877–4.915)
Marital status		0.808				0.535		
Unmarried/cohibiting (*n* = 118)	8 (6.8)		1	1	14 (11.9)		1	1
Married (*n* = 327)	21 (6.4)		0.944 (0.406–2.192)	1.422 (0.516–3.922)	32 (9.8)		0.806 (0.414–1.569)	0.531 (0.215–1.310)
Divorced/widowed (*n* = 96)	8 (8.3)		1.250 (0.451–3.464)	1.451 (0.444–4.740)	7 (7.3)		0.584 (0.226–1.511)	0.395 (0.272–3.323)
Transmission route		**0.007**				0.516		
HETs (*n* = 429)	22 (5.1)		1	1	43 (10.0)		1	1
IDUs (*n* = 96)	15 (15.6)		3.426 (1.704–6.887)*	1.623 (0.540–4.879)	9 (9.4)		0.929 (0.436–1.976)	0.518 (0.179–1.502)
MSM (*n* = 5)	0 (0)		0 (0)	0 (0)	1 (20.0)		2.244 (0.245–20.536)	18.519 (0.966–355.006)
Others/NA (*n* = 13)	0 (0)		0 (0)	0 (0)	0 (0)		0 (0)	0 (0)
CD4 + cell count (cells/ul)		0.127				0.259		
<200 (*n* = 229)	13 (5.7)		1	1	17 (7.4)		1	1
200–499 (*n* = 225)	14 (6.2)		1.102 (0.506–2.401)	0.803 (0.340–1.894)	26 (11.6)		1.629 (0.858–3.094)	1.406 (0.669–2.956)
≥500 (*n* = 83)	10 (12.0)		2.276 (0.957–5.411)	1.768 (0.652–4.794)	10 (12.0)		1.708 (0.749–3.899)	1.750 (0.640–4.787)

*^*a*^Chi-square and Fisher’s exact test. Statistically significant *P* values are indicated in bold. Of 543 clustering individuals, data on ethnicity, education, occupation, and CD4 + cell counts are missing for 4, 3, 2, and 6 patients, respectively. DRM, drug resistance mutation; PDR, pretreatment drug resistance; COR, crude odds ratio; AOR, adjusted odds ratio; CI, confidence interval; HETs, heterosexuals; IDUs, injection drug users; MSM, man who have sex with man; NA, not available.*

* indicates that the P value in logistic regression analysis is less than 0.05.

Regarding shared DRM, significant differences were found in sampling city, year of enrollment, subtype, gender, ethnicity, and occupation. As shown, participants from Baise city had a higher proportion of shared DRM than those from Qinzhou city (14.3 vs. 7.5%, *P* = 0.012; COR = 2.062, 95% CI = 1.165–3.650). However, this significance was lost in multivariate logistic regression analysis. A similar trend was also significant for females compared to males (14.5 vs. 7.9%, *P* = 0.021; COR = 1.965, 95% CI = 1.098–3.517) and Zhuang ethnicity compared to Han ethnicity (15.6 vs. 7.5%, *P* = 0.012; COR = 2.269, 95% CI = 1.277–4.031). Subtype CRF08_BC was more likely to have DRM sharing (COR = 6.673, 95% CI = 2.941–15.142; AOR = 8.641, 95% CI = 3.475–21.490). The lower limit of *E*-value of CRF08_BC and shared DRM was 3.133 (Supplementary Table 1).

## Discussion

In this cross-sectional study, we explored the DRM transmission dynamics and PDR prevalence among recently diagnosed and ART-naïve HIV-1 individuals with a relatively large sample size (*n* = 1,025) in the area that used to have a high incidence of drug abuse and HIV-1 infection in Guangxi.

Drug resistance testing for ART-naïve patients prior to the initiation of treatment has been reported to be cost-effective and potentially beneficial to patients (Weinstein et al., 2001; Luz et al., 2015). We observed a higher PDR prevalence (8.3%) in these two cities than the previously reported national average (6.8%) (Kang et al., 2020). One possible reason is that PDR in this region is mainly derived from subtype CRF08_BC commonly seen among IDUs before, and it has been found that IDUs are more prone to DR due to poor drug compliance and a variety of high-risk behaviors (Muyldermans and Sasse, 2014; Liu et al., 2019). Another potential reason might be that local DR is disseminated by individuals failing ART or with transmitted DRMs. Additionally, a long history of ART may also contribute to high PDR prevalence. However, the successful implementation of ART in Guangxi has controlled the regional PDR to a low level (Yang et al., 2019). Therefore, PDR prevalence in this region is moderate and below WHO’s 10% warning threshold (WHO, 2017). Since PDR may lead to virological failure, accumulation of additional DRMs, and increased regimen switching (Boender et al., 2015; Kityo et al., 2017), there is an urgent need for ongoing routine surveillance of PDR transmission dynamics.

Notably, PDR prevalence gradually declined within networks over time in this region. The increase in ART regimens, combined with refined knowledge and improved ART adherence (Thompson et al., 2012; Pennings, 2013), effectively reduced the prevalence of PDR, especially among patients diagnosed in the latter years of the study period. Moreover, considering that IDUs was significantly related to the increased PDR (Pham et al., 2015; Kang et al., 2020), the shift in transmission patterns from IDUs to HETs might also affect PDR prevalence in Guangxi. However, the proportion of shared DRM within networks significantly increased over time, indicating that sustained PDR surveillance in Guangxi should be strengthened to prevent the deterioration of DR.

We determined that NNRTI-related DRMs dominated the PDR prevalence among ART-naïve patients in this region, consistent with previous studies in Southwest China (Chen M. et al., 2012, 2018; Kang et al., 2020). In addition, HIV-1 strains with high levels of resistance to NNRTI were more prevalent than those to NRTI and PI, which may be related to mutations associated with decreased susceptibility to NNRTI that were generated rapidly in the early stages of the selection process with a low genetic barrier (Zhang et al., 2004). Furthermore, it is worth noting that the DRM V179D/E associated with NNRTI was the most common (Lu et al., 2017; Wang et al., 2019; Zhang et al., 2020), and spread widely within networks. Studies have reported that V179D/E has been on the rise among the MSM population in recent years (Li et al., 2016; Yin et al., 2019). Here, we found that sequences with V179D/E were distributed and networked among HETs, suggesting that V179D/E is involved in ongoing HIV-1 transmission in this region. Focusing on specific DRM connections within networks and then inferring possible transmission patterns between individuals might provide insights into HIV-1 intervention strategies.

This study found that the most prevalent HIV-1 genotype in this region was CRF08_BC, inconsistent with a previous study (Li et al., 2018). This finding may be related to the high prevalence of CRF08_BC in Baise city (Li et al., 2014), one of our sampling sites. Compared to CRF01_AE, HIV-1 strains subtyped as CRF08_BC were significantly correlated with DRM development. Clusters of the CRF08_BC subtype related to shared DRM were more frequent and larger than those of the CRF01_AE subtype. Moreover, PDR transmission and the DRM sharing patterns within networks in this region were both associated with the CRF08_BC subtype. Previous studies have found that CRF08_BC was one of the primary drivers of HIV-1 infection among IDUs, especially in Southwest China (He et al., 2012; Jiang et al., 2019). Frequent needle exchange and poor ART adherence among IDUs could lead to a higher risk of drug-resistant strains spreading in this population (Bangsberg et al., 2007; Thanh et al., 2009; Muyldermans and Sasse, 2014; Liu et al., 2019). Therefore, as two cities with a historically high incidence of drug abuse in Guangxi, the predominant HIV-1 subtype CRF08_BC in this region is more prone to DRMs and leads to widespread transmission, further emphasizing the necessity and urgency of strengthening routine PDR surveillance of CRF08_BC.

Similar to a previous study conducted in Fuyang, Anhui Province (Wu et al., 2019), we observed that ART-naïve patients over 50 years old were more likely to cluster within networks. The elderly are at higher risk of contracting HIV-1 compared to the general population in China (Wang et al., 2020) and Guangxi (2014). This observation could be attributed to many factors. First, older people tend to be locally settled and less mobile, so HIV-1 transmission among this subgroup is limited. Moreover, older men tend to have similar patterns of sexual behavior, such as being more likely to have commercial sex with local female sex workers (FSWs) or casual partners (Chen X. et al., 2012; Zhou et al., 2014). The geographic transmission hotspots formed by commercial HET contact between older men and FSWs significantly contributes to the local HIV-1 epidemic (Jiang et al., 2020). It has been reported that HIV-1 prevalence among elderly male clients of FSWs in Guangxi has continued to increase in recent years (Chen et al., 2016). Effective control measures, such as detecting TCs and developing targeted, and localized prevention strategies, should be given priority among the elderly.

This study had some limitations. First, we only recruited subjects from two cities (Baise and Qinzhou) in Guangxi, which may lead to selection bias. However, the results obtained from our relatively stable transmission network constructed with a large sample size were credible and could illustrate the transmission pattern of HIV-1 at least in these two cities since incomplete sampling may increase the chance of linking individuals who are not direct transmission partners in the network (Kusejko et al., 2018; Ragonnet-Cronin et al., 2019). Second, our risk factor assessments focused on limited factors and failed to assess the influence of certain drug use among IDUs, substance use among MSM, and sexual behaviors among HETs on PDR transmission and DRM sharing likelihood. Future molecular surveillance in Guangxi will greatly benefit from more detailed data.

In conclusion, this study demonstrates that the prevalence of PDR was moderate in this region. Sharing of specific DRMs (such as V106I and V179D/E) was frequent within networks, revealing the potential for widespread PDR dissemination in the future. Subtype CRF08_BC was more likely to have DRMs as well as shared DRMs and PDR transmission within the genetic network. Routine surveillance of PDR and strengthening control measures to prevent its development and dissemination are essential to guide the first-line ART regimens in Guangxi.

## Data Availability Statement

The datasets presented in this study can be found in online repositories. The names of the repository/repositories and accession number(s) can be found below: https://www.ncbi.nlm.nih.gov/genbank/, accession numbers: MH789749, MH 789754, MH 789759, MH 789766, MH 789775, MH 789788, MH 789810, MH 789811, MH 789832, MH 789841, MH 789843, MH 789852, MH 789857, MH 789858, MH 789868, MH 789871, MH 789872, MH 789874, MH 789878, MH 789890, MH 789892, MH 789915, MH 789920, MH 789924, MW867330–MW868165, and MZ269529–MZ26969.

## Ethics Statement

The studies involving human participants were reviewed and approved by the Human Research Committee of Guangxi Medical University (Ethical Review No. 20170228-21). The patients/participants provided their written informed consent to participate in this study.

## Author Contributions

FZ, BL, LY, and HaL designed and conceived this research, and wrote the manuscript. YuY, YaY, HuL, SZ, CQ, and JuJ performed the experiments, analyzed the data, and prepared the figures and tables. XL, ZL, NL, JiJ, JH, and RH provided insight into the experimental design and data analysis. All authors read and approved the final manuscript.

## Conflict of Interest

The authors declare that the research was conducted in the absence of any commercial or financial relationships that could be construed as a potential conflict of interest.

## Publisher’s Note

All claims expressed in this article are solely those of the authors and do not necessarily represent those of their affiliated organizations, or those of the publisher, the editors and the reviewers. Any product that may be evaluated in this article, or claim that may be made by its manufacturer, is not guaranteed or endorsed by the publisher.

## Footnotes

1
https://www.hiv.lanl.gov

2
https://comet.lih.lu

3
http://jphmm.gobics.de

4
https://hivdb.stanford.edu/
