# Supplementary material for: Using Molecular Transmission Networks to Reveal the Epidemic of Pretreatment HIV-1 Drug Resistance in Guangxi, China
Source: Front Genet. 2021 Sep 10;12:688292. doi: 10.3389/fgene.2021.688292 (PMC8460771; doi:10.3389/fgene.2021.688292)
Supplement: Supplementary file 1 [file Table_1.docx]

Table S1 Sensitivity analysis of unmeasured confounders.

| Dependent variable | Independent variable | AOR (95% CI) | E-values |
| --- | --- | --- | --- |
| DRM | CRF08_BC subtype | 2.349(1.593-3.463) | 2.436 |
| Clustering | Age >50 years old | 2.251(1.366-3.710) | 2.367 |
| PDR | CRF08_BC subtype | 4.083(1.498-11.127) | 3.457 |
| Shared DRM | CRF08_BC subtype | 8.641(3.475-21.490) | 5.327 |
| Abbreviation: DRM, drug resistance mutation; PDR, pretreatment drug resistance; AOR, adjusted odds ratio; CI, confidence interval. | | | |
